# Streptozotocin Impairs Proliferation and Differentiation of Adult Hippocampal Neural Stem Cells *in Vitro*-Correlation With Alterations in the Expression of Proteins Associated With the Insulin System

**DOI:** 10.3389/fnagi.2018.00145

**Published:** 2018-05-18

**Authors:** Ping Sun, Gabriela Ortega, Yan Tan, Qian Hua, Peter F. Riederer, Jürgen Deckert, Angelika G. Schmitt-Böhrer

**Affiliations:** ^1^Key Laboratory of Molecular Target & Clinical Pharmacology, School of Pharmaceutical Science & The Fifth Affiliated Hospital, Guangzhou Medical University, Guangzhou, China; ^2^Center of Mental Health, Department of Psychiatry, Psychosomatics, and Psychotherapy, University Hospital of Würzburg, Würzburg, Germany; ^3^School of Preclinical Medicine, Beijing University of Chinese Medicine, Beijing, China

**Keywords:** streptozotocin, neural stem cells, proliferation, differentiation, insulin-like growth factor 1 receptor, insulin receptor, glucose transporter, Alzheimer’s disease

## Abstract

Rats intracerebroventricularily (icv) treated with streptozotocin (STZ), shown to generate an insulin resistant brain state, were used as an animal model for the sporadic form of Alzheimer’s disease (sAD). Previously, we showed in an *in vivo* study that 3 months after STZ icv treatment hippocampal adult neurogenesis (AN) is impaired. In the present study, we examined the effects of STZ on isolated adult hippocampal neural stem cells (NSCs) using an *in vitro* approach. We revealed that 2.5 mM STZ inhibits the proliferation of NSCs as indicated by reduced number and size of neurospheres as well as by less BrdU-immunoreactive NSCs. Double immunofluorescence stainings of NSCs already being triggered to start with their differentiation showed that STZ primarily impairs the generation of new neurons, but not of astrocytes. For revealing mechanisms possibly involved in mediating STZ effects we analyzed expression levels of insulin/glucose system-related molecules such as the glucose transporter (GLUT) 1 and 3, the insulin receptor (IR) and the insulin-like growth factor (IGF) 1 receptor. Applying quantitative Real time-PCR (qRT-PCR) and immunofluorescence stainings we showed that STZ exerts its strongest effects on GLUT3 expression, as GLUT3 mRNA levels were found to be reduced in NSCs, and less GLUT3-immunoreactive NSCs as well as differentiating cells were detected after STZ treatment. These findings suggest that cultured NSCs are a good model for developing new strategies to treat nerve cell loss in AD and other degenerative disorders.

## Introduction

Streptozotocin (STZ) is a glucosamine derivative of nitrosourea produced by *Streptomyces achromogen*s and toxic to the insulin-producing β cells of the pancreas in mammals (Eileen Dolan, 1997). It is used in medicine for treating certain cancers of the Islets of Langerhans (Murray-Lyon et al., 1968; Brentjens and Saltz, 2001) and also used in research to produce animal models for type 1 diabetes mellitus (T1DM) via high dose intraperitoneal (i.p.) injections (Like and Rossini, 1976) and type 2 diabetes mellitus (T2DM) with low doses i.p. injections (Reaven and Ho, 1991; Wang and Gleichmann, 1998; Yuan et al., 2016). STZ treatment causes typical aging-associated changes, such as telomere instability (Paviolo et al., 2015), mitochondrial dysfunction (Raza and John, 2012), genomic instability (Attia et al., 2009), metabolic dysfunction (Rodríguez-Mañas et al., 2009) and cellular senescence (Oubaha et al., 2016). Furthermore, T2DM induced by STZ i.p. injections exhibit increased brain aging and AD-like pathology, such as brain atrophy, Aβ aggregation, and synapse loss (Wang et al., 2014). With moderate to low dosage administration, STZ causes insulin resistance by decreased autophosphorylation of the insulin receptor (IR; Kadowaki et al., 1984; Blondel and Portha, 1989).

IR can be activated by insulin-like growth factor (IGF)-1 and -2 besides insulin (Ward and Lawrence, 2009) and plays a key role in the regulation of glucose homeostasis. Besides the insulin-sensitive glucose transporter (GLUT)4, which is shown to be primarily expressed by cerebellar neurons, the insulin-independent GLUT1 is the main transporter responsible for glucose transport across the blood-brain barrier into the brain and into astrocytes (Leybaert et al., 2007) and GLUT3 is responsible for glucose transport into neurons (for review, see Simpson et al., 2007). Reduced IR signaling may finally results in insulin resistance, accompanied by impaired ability to maintain cell glucose and energy homeostasis (Draznin, 2006; Moloney et al., 2010; Talbot et al., 2012; Talbot, 2014). Insulin resistance in non-neural tissues can be considered as the reason for many peripheral metabolic disorders such as T2DM (Reaven, 1988). Insulin resistance in the brain may trigger pathophysiological key events of neurodegenerative disorders such as Alzheimer’s disease (AD) and therefore can be linked to these disorders (Correia et al., 2011; Talbot et al., 2012).

Individuals with diabetes have higher risk for developing dementia syndromes (Ott et al., 1999; Roriz-Filho et al., 2009; Riederer et al., 2017). Epidemiologic studies revealed a significant association between T2DM and AD, and that metabolic dysfunctions like hyperglycemia and hyperinsulinemia and/or hypoinsulinemia are closely correlated with AD pathophysiology (Ryan and Geckle, 2000; Allen et al., 2004; Matsuzaki et al., 2010; Ou et al., 2018). Therefore, disturbances of brain glucose uptake, glucose tolerance and glucose utilization and impairment of the insulin/IR signaling cascade are thought to be key targets for the neuropathology of the sporadic form of AD (sAD) which covers >95% of AD patients (Grünblatt et al., 2007; de la Monte, 2009; Salkovic-Petrisic et al., 2009; Talbot et al., 2012).

STZ intracerebroventricularily (STZ icv) treated rats, which develop an insulin resistant brain state shortly after treatment, have been suggested to act as an animal model for sAD (Grünblatt et al., 2007; de la Monte, 2009; Salkovic-Petrisic et al., 2009). In this animal model, cognitive function was shown to be impaired already a few weeks after STZ icv treatment (Mayer et al., 1990; Blokland and Jolles, 1993) paralleled by cholinergic deficits (Hellweg et al., 1992), oxidative stress (Sharma and Gupta, 2001), neuronal loss (Shoham et al., 2003), amyloid angiopathy (Salkovic-Petrisic et al., 2011), increasing tau protein (Salkovic-Petrisic et al., 2006) as well as insulin signaling pathway damage (Salkovic-Petrisic et al., 2006). Brain glucose metabolism has been found to be dramatically harassed in this animal model including decreased glucose utilization (Duelli et al., 1994) and reduced glycolytic key enzymes activity (Plaschke and Hoyer, 1993), diminished adenosine triphosphate (ATP) and creatine phosphate (Lannert and Hoyer, 1998). The neuron-specific GLUT3 has been found to be significantly reduced in this rat model (Salkovic-Petrisic et al., 2013).

Neural stem cells (NSCs), which were able to give birth to new neurons, astrocytes and oligodendrocytes in the developing as well as in the adult brain, primarily exist in few neurogenic regions in most mammals, including humans (Altman and Das, 1965; Eriksson et al., 1998; Wiskott et al., 2006). Aside from the subventricular zone of the lateral ventricle, the dentate gyrus (DG) of the hippocampus is one of the neurogenic regions in the adult brain (Kempermann and Gage, 2000; Kempermann et al., 2004; Ming and Song, 2005; Von Bohlen Und Halbach, 2011). From a functional point of view, hippocampal adult neurogenesis (AN) plays an important role in structural plasticity and network adaptation and is likely to contribute to learning and memory processes (Aimone et al., 2010). The question of a possible involvement of altered AN in AD etiopathology is not satisfactorily answered, yet. Discrepancies between results of various studies may result from the different AD animal models used in these AN studies. Besides, NSCs can be discussed to have therapeutic potential in the treatment of neurodegenerative disorders such as AD (Abdel-Salam, 2011), Parkinson’s disease (Nishimura and Takahashi, 2013), as well as traumatic injuries of the nervous system (Longhi et al., 2005) and aging (Leeman et al., 2018). Hippocampal NSCs transplantation as well as stimulating AN through physical exercise and drugs could rescue cognitive deficits in AD mice (van Praag et al., 1999; Dong S. et al., 2012; Chen et al., 2015) by enhancement of hippocampal synaptic density (Blurton-Jones et al., 2009). Elevating AN may have therapeutic potential for the treatment of AD, e.g., compensation of neuronal as well as synaptic loss observed in the AD brain (Selkoe, 2002). Therefore, it is worth characterizing NSCs and studying their performance and regulation during normal aging as well as in the disease state.

AN is a dynamic process tightly regulated by intrinsic and extrinsic factors, including molecules of the Insulin/IGF-1 signaling pathway (Bateman and McNeill, 2006). IGF-1 is a key factor in the regulation of NSCs, as in the absence of IGF-I neither the epidermal growth factor (EGF) nor the fibroblast growth factor 2 (FGF-2) were able to induce the proliferation of E14 mouse striatal cells (Arsenijevic et al., 2001). Moreover, high concentrations of insulin promote the differentiation of newborn cells into neurons (Han et al., 2008). However, the impact of the Insulin/IGF-1 signaling pathway on the proliferation of NSCs and the differentiation fate of their progeny has not been uncovered at the cellular level. Our recently published *in vivo* study dealing with the effects of STZ icv injections on AN indicated reduced neuron generation after 3 months predominantly in the septal part of the hippocampus (Sun, 2015; Sun et al., 2015).

Therefore we aimed at uncovering cellular mechanisms underlying the negative effect of STZ on AN. With an *in vitro* approach using hippocampal NSCs we investigated the possible influence of STZ on the proliferation of NSCs, their migration and differentiation, and whether STZ treatment alters the expression levels of genes related to the insulin system such as the IR, IGF-1 receptor (IGF-1R) and GLUT1 and 3.

## Materials and Methods

### Isolation of Neural Stem Cells—Establishment of Primary Adult Neural Stem Cell Cultures of Rat Hippocampi

Adult NSCs were derived from both hippocampi of Wistar rats (in total about 50 rats were used, 2 months ± 1 week old; Charles River, Sulzfeld, Germany). After performing a pilot study using rats of different ages with the result that younger animals generate more neurospheres than older ones we decided to continue working with these young adult rats, even if older animals would have been the better choice to study neurobiological mechanisms of human sAD with an onset around 65 years. In brief, hippocampi were dissected mechanically on ice and enzymatically dissociated in a 0.01% papain–0.1% protease–0.01 DNase I (PPD) solution (each enzyme was obtained from Worthington Biochemicals, USA and dissolved in Hank’s Balanced Salt Solution). Cells were collected by centrifugation at 110 *g* for 7 min (RT) and then re-suspended in proliferation cell culture medium composed of NeuroCult™ NS-A Basal medium (containing 0.6% glucose; STEMCELL_Technologies, USA) supplemented with Neurocult™ NS-A proliferation supplement (containing 25 μg/ml insulin; 10%), EGF (20 ng/ml, Peprotech, Germany), basic fibroblast growth factor (bFGF; 10 ng/ml, Peprotech, Germany) and Heparin (2 μg/ml, STEMMCELL, USA). Next, cells were plated onto T25 culture flasks (Corning, USA) and maintained in a humidified incubator with 5% CO_2_ at 37°C. In general, proliferation medium was replaced every 7 days. After 2 days of incubation in proliferation medium neurospheres had been formed and were visible.

For the characterization of cells composing such neurospheres immunofluorescence stainings were performed using antibodies detecting nestin, a marker for NSCs. For that, neuroshperes were seeded on poly-L-ornithine/laminin-coated coverslips (Neuvitro, El Monte, CA, USA) in proliferation culture medium. After approximately 2 h of incubation, most neurospheres were attached to the coverslips, a prerequisite for the subsequent immunofluorescence staining. Then, they were fixed with 4% PFA (dissolved in PBS) at RT for 20 min and immunostained for nestin (for details see below).

### Treatment With STZ

#### Stem Cell Proliferation

First, a dilution series of STZ was applied to NSCs to select a suitable STZ concentration. For that, neurospheres (which had been passaged already two times) were enzymatically dissociated using a PPD solution and then obtained single cells were seeded into 96-well plates (Life Technologies, Gaithersburg, MD, USA) with 2000 cells per well in proliferation cell culture medium (see above). A 0.5 M stock solution of STZ diluted in citrate buffer (0.1 M, pH 4.5) was prepared. Cells were incubated in proliferation medium containing five different final STZ concentrations (0, 1.0, 2.5, 5.0 and 10 mM) for 4 days. Then, the number of neurospheres per well (size >5 cells) was estimated under the BX40 microscope (Olympus, Tokyo, Japan) at 10× magnification.

In subsequent experiments (proliferation, migration and differentiation assays) STZ at a concentration of 2.5 mM was used with different incubation times (2–8 days).

#### Time-Dependency of STZ Influence

For unraveling the time-dependency of STZ effects on the generation of neurospheres, we performed a long-term incubation study with or without 2.5 mM STZ. The same proliferation assay was performed as described above and the number of neurospheres per well was estimated after 2, 4, 6 and 8 days of incubation with 2.5 mM STZ in proliferation cell culture medium under the microscope. Three independent experiments with each experiment stemming from three biological replicates (using the hippocampus of three rats) were performed, but only the results of one experiment were presented.

#### Effect of STZ on the Size of Newly Generated Neurospheres

For the evaluation of the *percentage of neurospheres of different sizes*, we measured the diameter of each neurosphere (μm). Neurospheres were generated using a single cell suspension (generated via dissecting neurospheres as described above) and then plated in a 96-well plate (2000 cells/well) for 7 days. The number of neurospheres exhibiting certain sizes (<50 μm, 50–99 μm, 100–149 μm, 150–199 μm and ≥200 μm) were estimated with the help of the BX40 microscope and the Image-Pro-Plus 5.0 software (Media Cybernetics, Rockville, MD, USA). We performed these experiments three times, collected all the data and then calculated the mean size ± SD of neurospheres with a certain size.

#### 5-Bromodeoxyuridine (BrdU) Incorporation Assay

For the estimation of the proliferation of NSCs a BrdU incorporation assay was performed. For that, cells of a single cell suspension were generated via dissecting neurospheres (as described above) and were then seeded on pre-coated coverslips in a 24 well plate (10,000 cells per well; Sarstedt, Nümbrecht, Germany). After 2 days of incubation in proliferation medium, 10 μM BrdU (BrdU stock solution contained 10 mM BrdU dissolved in D-PBS) was added to culture medium for 4 h to label proliferating cells. Next, cells were fixed with 4% PFA and a BrdU/DAPI immunofluoresence double staining was performed (for details see below). After picturing of stained cells the percentage of BrdU-positive cells was calculated using 10–15 pictures per group out of three independent experiments.

#### Migration Assay

Rat hippocampal neurospheres were collected via centrifugation (110 *g*, 7 min) and then re-suspended with differentiation cell culture medium composed of Neurocult™ NS-A basal medium (with 0.6% glucose) and 10% Neurocult™ NS-A differentiation supplement (with 25 μg/ml insulin). Neurospheres were seeded on pre-coated coverslips in a 24-well plate (10–14 neurospheres per well; Sarstedt, Nümbrecht, Germany) and after approximately 2 h (most of the neurospheres should have been attached in the meantime) the differentiation cell culture medium was refreshed with culture medium with or without 2.5 mM STZ. After 2 days of incubation, images were taken using the BX40 microscope and the migration distance was estimated with the help of Image-Pro-Plus 5.0 software. Distance from the cell to the rim of the respective originating neurosphere was defined as the migration distance. Overall, 15–20 images per group out of three independent experiments were analyzed.

#### Differentiation Assay

For characterization of the cellular phenotype of differentiating newborn cells and the effect of STZ on the differentiation fate, 10–15 neurospheres per well after the second passage were collected and then promoted to differentiate using the same procedure as described in the migration assay above. After 2 days of incubation, we aspired cell culture medium and added 0.5 ml of the fixative (4% PFA dissolved in PBS) for 30 min at RT. Immunofluoresence detection of young neurons and astrocytes with anti-Tuj-1 and GFAP antibodies, respectively, was performed. Finally, a DAPI stain was applied for visualizing of all cultured cells. For quantification of immunoreactive (ir) cells, 15–20 images out of three independent experiments was determined.

#### Detection of Insulin Receptor and Glucose Transporter 3 Protein in NSCs as Well as in Differentiating Cells

For immunostaining of NSCs, single cells enzymatically dissociated from neurospheres were placed on pre-coated coverslips in a 24-well plate (20,000 cells/well). After exposure to 2.5 mM STZ for 2 days, we fixed obtained cells with 4% PFA and immunostained them with antibodies detecting the IR and the glucose transporter 3 (GLUT3; for details see below).

In order to further characterize differentiating cells, they were generated (as described above in the chapter “differentiation assay”; also with or without 2.5 mM STZ) and processed for immunofluorescence stainings with GLUT3 and IR antibodies (for details see below). A DAPI stain was applied for visualizing all cultured cells. For quantification, we calculated the percentage of IR or GLUT3 positive cells using 15–20 replicate images per group out of three independent experiments.

### Single and Double Immunofluorescence Staining

#### Immunodetection of BrdU

After NSCs had been fixed with 4% PFA (dissolved in PBS), they were washed three times with TBS for 5 min and DNA denaturation was achieved by incubation with 1 N HCl at 37°C for 10 min. Then, we neutralized low pH-values with 0.1 M boric acid (pH 8.5) for 10 min and subsequently rinsed the cells with TBS three times. Non-specific immunoreactions were blocked with 5% normal goat serum for 1.5 h. The mouse anti-BrdU antibody (monoclonal antibody, 1:300; MCA2483; Serotec, Kidlington, UK) was used as the primary antibody, and donkey anti-mouse IgGs conjugated with Alexa 555 (1:600; Life science, Carlsbad, CA, USA) as the secondary antibody. After rinsing remaining cells fixed to coverslips, all nuclei were stained with DAPI (300 nM) for 5 min at RT, and then washed again. Finally, coverslips were mounted to slides with Fluoromount™Aqueous Mounting Medium (DAKO, Hamburg, Germany).

#### Single and Double Immunofluorescent Stainings

For immunofluorescent single and double stainings of NSCs as well as differentiating cells with primary antibodies detecting Tuj-1 (monoclonal mouse antibody, 1:250; ab14545, Abcam, Cambridge, MA, USA), GFAP (polyclonal rabbit antibody, 1:500; Z0334, DAKO, Hamburg, Germany), Nestin (polyclonal rabbit antibody, 1:250; ab92391, Abcam, Cambridge, MA, USA), IR (monoclonal mouse antibody, 1:250; ab69508, Abcam, Cambridge, USA) and GLUT3 (polyclonal rabbit, 1:500; ab41525, Abcam, Cambridge, USA) proteins, applied protocols were similar to the BrdU staining described above, but without treatment with 1 N HCl for 10 min and subsequent incubation step with boric acid. For double immunofluorescent stainings we used two primary antibodies produced in different species. Secondary antibodies utilized were the following: donkey anti mouse IgGs conjugated with Alexa 488 and donkey anti-rabbit IgGs conjugated with Alexa 555 (both diluted 1:500, Life science, Carlsbad, CA, USA). DAPI was always used as a nuclear stain.

### Quantitative Real Time-PCR

Total RNA was extracted from neurospheres, which had been treated with or without 2.5 mM STZ for 2 days, using RNeasy kit from QIAGEN following manufacturer’s instructions. cDNAs were synthesized with the help of the first cDNA synthesis kit (Bio-Rad, Hercules, CA, USA) and 20 ng RNA per sample. QRT-PCR was performed in 384-well plates (life technologies, Gaithersburg, MD, USA) using a CFX384 Real-Time system (Bio-Rad, USA) and SYBR green. The relative amount of the message of interest was normalized to the expression level of the reference gene GAPDH. C_T_ values of duplicates or triplicates were analyzed with LinRegPCR software.

### Statistical Analysis

For the NSCs proliferation assay and the cell migration study with various concentrations of STZ, data analyses were performed with one-way ANOVA, followed by *post hoc* comparison with Bonferroni *post hoc* test using SPSS software (Version 22.0, IBM Inc., Chicago, IL, USA). For the statistical evaluation of all other experimental data, Student’s *t*-test was used. Data were presented as mean ± standard deviation (SD). Significance levels were set at *p < 0.05; **p < 0.01; ***p-value < 0.001.

## Results

**Figure 1:**
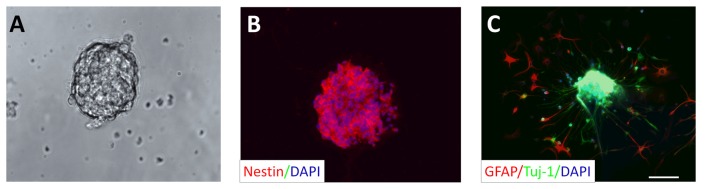
Neural stem cell (NSC) culture: neurosphere generation derived from adult rat hippocampus. **(A)** Culturing of NSCs in proliferation cell culture medium resulted in the formation of neurospheres after 1 week. **(B)** Representative neurosphere after the second passage shows immunoreactivity for nestin, a marker for NSCs (red). All cells composing the neurosphere were counterstained with the cell nucleus stain DAPI (blue). **(C)** Cells originating in a neurosphere, but cultivated in differentiation culture medium for 2 days display positive immunoreactivity for the marker for immature neurons Tuj-1 (green) and the astrocytic marker GFAP (red). DAPI was used to stain all cell nuclei (blue). Scale bar in **(C)** represents 50 μm for **(A–C)**.

We cultured NSCs using the three-dimensional neurosphere method. NSCs isolated from adult rat hippocampus proliferated quickly forming small free-floating clusters of NSCs first, and then forming larger neurospheres 1 week after seeding (Figure 1A). Nestin, a marker protein of neural progenitor cells, was expressed in these neurospheres (Figure 1B). After replacing the proliferation cell culture medium by the differentiation cell culture medium [without EGF and bFGF], neurospheres were cultivated for two additional days. Cells originating from such a neurosphere started to migrate and became immunoreactive for Tuj-1 (marker for immature neurons) or GFAP (marker for astrocytes; Figure 1C).

### STZ Impairs the Proliferation of Neural Stem Cells

**Figure 2:**
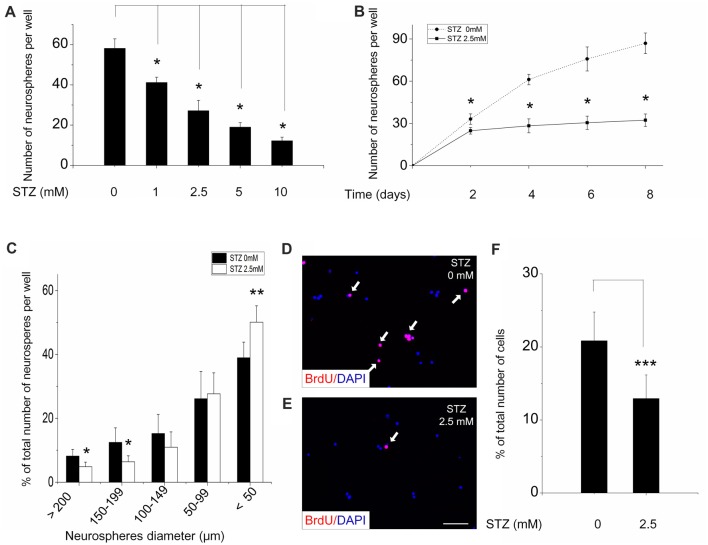
Streptozotocin (STZ) decreases proliferation of neural stem cells and the formation of neurospheres. Cells from neurospheres after the second passage were incubated with various concentrations of STZ for 4 days **(A)** and with 2.5 mM STZ for 8 days **(B)**. **(C)** Size distribution of neurospheres after incubation in culture medium with or without 2.5 mM STZ for 7 days. A significantly lower percentage of large neurospheres was counted in culture media with STZ. **(D–F)** STZ decreases the number and percentage of bromodeoxyuridine (BrdU)-positive NSCs. Data in **(A,B)** are presented as the number of neurospheres per well ± standard deviation (SD). Data in **(C)** are expressed as the percentage of neurospheres of a certain size (% of total number of neurospheres) ± SD. Data in **(F)** are presented as the percentage of BrdU-positive cells (% of total number of DAPI-positive cells) ± SD. *p-value < 0.05, **p-value < 0.01, ***p-value < 0.001.

To determine the optimal STZ concentration for investigating its effect on the proliferation of NSCs *in vitro*, NSCs (single cells, after the second passage) were seeded in a 96-well plate and treated with increasing concentrations of STZ. Four days of incubation with different STZ concentrations resulted in an overall, but dose-dependent, decrease of the number of neurospheres (ANOVA: *p* < 0.001; *Post hoc* analysis: 1 mM: 29.3%, *p* < 0.05; 2.5 mM: 53.3%, *p* < 0.05; 5 mM: 67.3%, *p* < 0.05 and 10 mM: 79.0%, *p* < 0.05) compared to the control group (0 mM STZ; Figure 2A).

As we preferred moderate, but not too strong effects of STZ, we choose 2.5 mM for all subsequent experiments. To determine the time-dependency of the effectiveness of STZ, NSCs were treatment with STZ for different time periods and the number of neurospheres was then counted (Figure 2B). STZ significantly decreased the number of neurospheres in a time-dependent manner (ANOVA: *p* < 0.05; *Post hoc* analysis: after 2 days of incubation a reduction of 25.1%, with *p* < 0.05; after 4 days a reduction of 53.85%, *p* < 0.05; after 6 days a reduction of 59.8%, *p* < 0.05; after 8 days a reduction of 62.8%, *p* < 0.05). As it is known that the size of neurospheres is directly related to proliferative capacity, we quantified the diameter of neurospheres after 7 days in the presence or absence of STZ. In cell culture medium with STZ significantly lower numbers of big neurospheres, e.g., with a diameter of ≥200 μm (*p* = 0.018, 4.85 ± 1.43%) and of neurospheres with a diameter between 150 μm and 199 μm (*p* = 0.025, 6.41 ± 1.86%) were detected when comparing to neurospheres in cell culture medium without STZ (≥200 μm: 8.21 ± 2.10%; 150–199 μm: 12.48 ± 4.45%). However, significantly greater number of small neurospheres with a diameters <50 μm were detected in the STZ treatment group (*p* = 0.007, 50.10 ± 5.10%) compared to control group (38.96 ± 4.90%; Figure 2C).

The thymidine analog bromodeoxyuridine (BrdU) is widely used to label cell proliferation because it incorporates into replicating DNA of dividing cells and can be immunodetected subsequently (Taupin, 2007). Without STZ treatment about 20% of NSCs had incorporated BrdU after 4 h of incubation (Figure 2F). Adding STZ to the cell culture medium resulted in a significant decrease of the percentage of BrdU-positive cells out of the total number of DAPI-positive cells (*p* < 0.001) to 12.9% (Figures 2D–F).

### STZ Does Not Affect the Migration of Newborn Differentiating Cells

**Figure 3:**
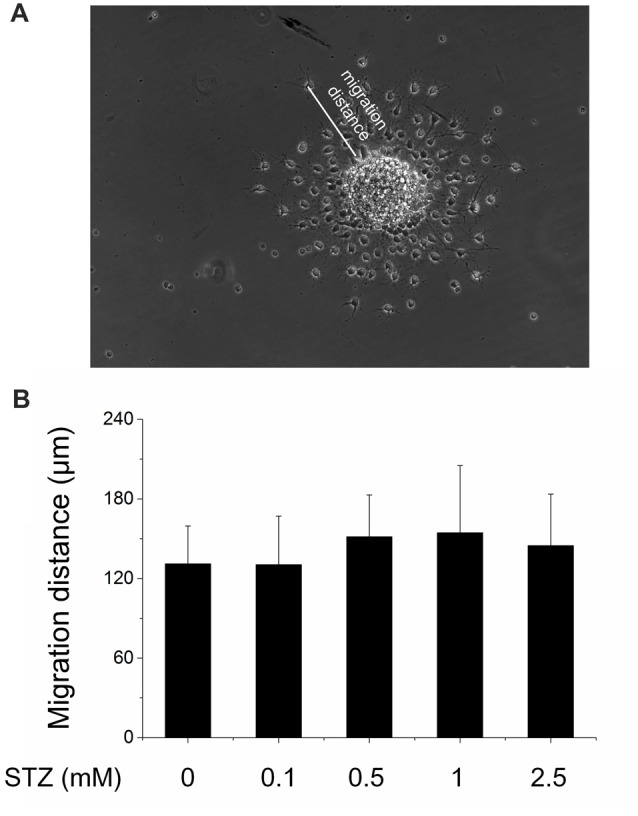
No effect of STZ on the migration capability of newborn differentiating cells. Neurospheres after the second passage were seeded in 24-well plates (10–15 neurospheres) and exposed to different concentrations of STZ in differentiation culture medium. **(A)** Representative image of migrating cells originating from a neurosphere. Scale bar represents 100 μm. **(B)** Migration distances of cells originating from neurospheres exposed to 0.0, 0.1, 0.5, 1.0 and 2.5 mM STZ. Data are expressed as the mean migration distance measured from the differentiating cells to the rim of the neurosphere ± SD.

In the subgranular zone of the hippocampal DG, NSCs differentiate into immature neurons which then migrate into the granule cell layer, where they mature into granule cells finally integrating into local neuronal circuitries (Ming and Song, 2005). To study the effect of STZ on this migration process, 10–15 neurospheres after the second passage were directly plated on pre-coated coverslips in 24-well plates and incubated in differentiation culture medium with or without STZ (0.1, 0.5, 1, 2.5 mM STZ) for 2 days. The migration distance of newborn cells treatment with STZ were very similar with the control group, which suggested that the STZ-exposure did not affect the ability and speed of newborn cells to migrate (ANOVA; *p* = 0.215; Figure 3).

### Effect of STZ on the Differentiation Fate of Newborn Cells

**Figure 4:**
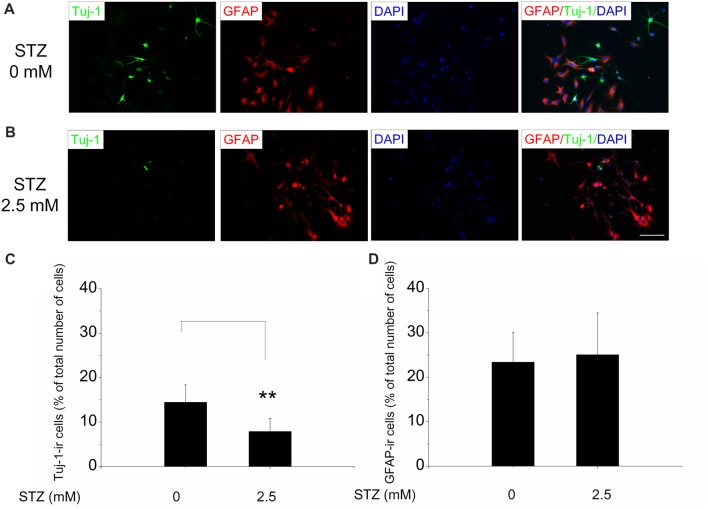
STZ diminishes the generation of new neurons, but has no effect on the number of new astrocytes. Neurospheres after the second passage were exposed to 2.5 mM STZ for 2 days in differentiation medium, and then stained with antibodies. **(A,B)** Representative photomicrographs of early differentiating cells with Tuj-1 (green) and GFAP (red) immunoreactivity exposed to 0 mM **(A)** or 2.5 mM STZ **(B)** for 2 days. All cells were counterstained with DAPI (blue). Scale bar represents 50 μm. **(C,D)** Quantitative analysis of the percentage of Tuj-1- or GFAP-immunoreactive (ir) cells analyzing 10–12 representative images per treatment group. Data are expressed as the percentage of Tuj-1- **(C)** or GFAP- **(D)** -ircells (in relation to the overall number of DAPI-positive cell nuclei) ± SD. **p-value < 0.01.

The neuronal phenotype of differentiating cells was determined by using Tuj-1 as a marker of immature neurons and GFAP as a marker for astrocytes. After 2 days of incubation in the differentiation medium, Tuj-1- and GFAP-positive cells were found in close vicinity of the neurospheres they originate from (Figure 1C). Double immunofluorescence stainings of cells, differentiated for 2 days in cell culture medium with and without STZ, suggested a negative effect of STZ exclusively on the number of Tuj1-positive cells, but not on the number of GFAP-positive cells (Figures 4A,B). Quantitative analysis demonstrated that the percentage of cells immunoreactive for Tuj-1 were significantly lower after 2 days of STZ treatment than those incubated in the normal differentiation medium (a decrease of 45.5%; *p* = 0.003; Figure 4C). However, the percentages of cells immunopositive for GFAP were not significantly different between the STZ treatment and the control group (Figure 4D).

### Effect of STZ on Insulin Receptor, Insulin-Like Growth Factor 1 Receptor and Glucose Transporter 1 and 3 mRNA Expression in Neural Stem Cells

**Figure 5:**
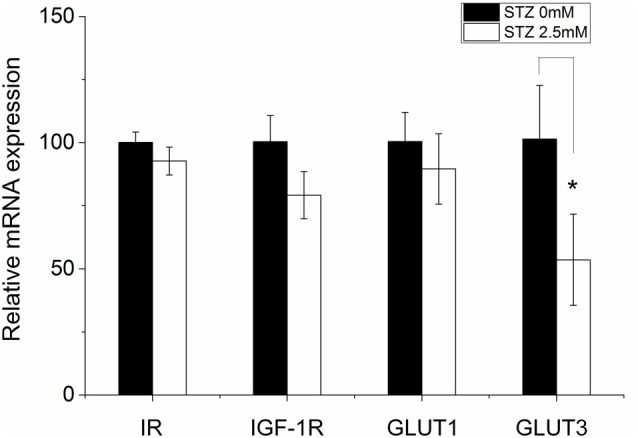
STZ impacts the expression of some metabolism-related genes in neural stem cells. Ten to fifteen neurospheres after the second passage were seeded in a 6-well plate exposed to either 0.0 or 2.5 mM STZ for 2 days. Then, cells were collected and analyzed via quantitative real-time PCR. The relative mRNA expression level of glucose transporter (GLUT) 3 is significantly reduced in neural stem cells (NSCs) by 46.4% (*p* = 0.041) in consequence of 2.5 mM STZ exposure. Expression differences of the insulin receptor (IR), insulin-like growth factor 1 receptor (IGF-1R), and GLUT1 did not reach statistical significance. Data are presented as the mean of relative mRNA expression levels ± SD; *p-value < 0.05.

In the STZ icv treatment rat model (*in vivo*), brain glucose/energy metabolism abnormalities were found in all hippocampal subfields, such as decreased glucose utilization (Duelli et al., 1994). GLUT3 is most known for its specific expression in neurons and has originally been designated as the neuronal GLUT (Kayano et al., 1988) and also been found to be expressed in adult NSCs (Maurer et al., 2006). Furthermore, insulin system dysfunction accompanied by diminished IR expression in hippocampus develops in consequence of STZ-icv administration (Grünblatt et al., 2007; Salkovic-Petrisic et al., 2013). In order to study possible STZ effects on the expression of glucose metabolism-related genes such as IR, IGF-1R, GLUT1 and GLUT3, we performed quantitative real time-PCR (qRT-PCR). After 2 days of incubation in cell proliferation medium, STZ remarkably decreased the relative expression levels of GLUT3 mRNA by 46.4% (*p* = 0.041). However, STZ treatment did not affect relative expression levels of IR and GLUT1 in NSCs (Figure 5).

### Effect of STZ on the Expression of Insulin Receptor and Glucose Transporter 3 Protein Levels in NSCs and Differentiating Cells

**Figure 6:**
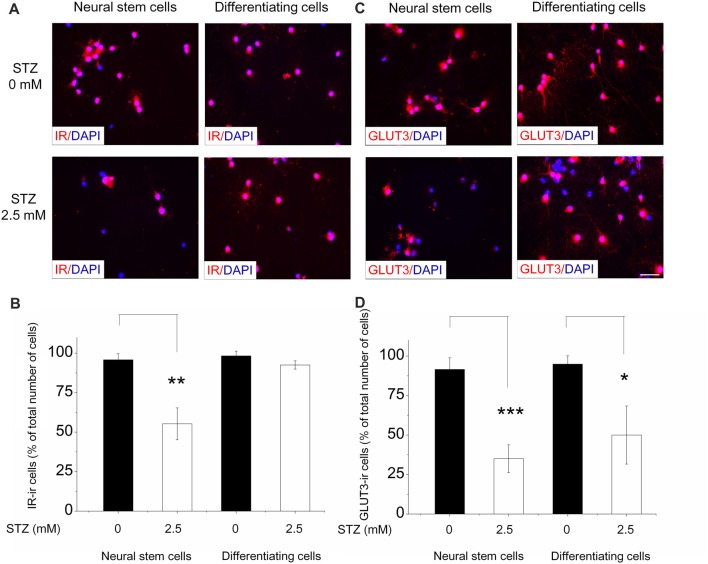
STZ affects IR and GLUT 3 protein levels in neural stem cells and differentiating cells differentially. For the analysis of neural stem cells (NSCs), neurospheres were dissociated and seeded in a 24-well plate (20,000 cells/well). After 2 days of 0.0 or 2.5 mM STZ exposure we immunostained them for the detection of IR and Glucose transporter 3 (GLUT3). For differentiating cells, neurospheres were seeded in a 24-well plate and let them grow in differentiation culture medium with or without 2.5 mM STZ for 2 days. **(A,C)** Immunostaining with IR and GLUT3 antibodies revealed that almost all NSCs and differentiating cells express IR as well as GLUT3. **(B)** STZ treatment resulted in a lower percentage of IR-immunoreactive (ir) NSCs, but not of differentiating cells. **(D)** STZ treatment affects GLUT3 protein expression in NSCs as well as in differentiating cells. Scale bar in **(C)** represents 20 μm for all images. Data in **(B,D)** are expressed as the percentage of IR- or GLUT3-ir cells (in relation to the overall number of DAPI-stained cell nuclei) ± SD. *p-value < 0.05, **p-value < 0.01, ***p-value < 0.001.

We further studied the effects of STZ on IR and GLUT3 protein expression levels in NSCs and differentiating cells via immunostaining with respective antibodies and used DAPI for counterstaining. For immunostaining of NSCs, single cells dissociated from neurospheres were plated on pre-coated coverslips in proliferation medium with and without 2.5 mM STZ for 2 days. For differentiating cells, neurospheres were seeded on pre-coated coverslips in differentiation culture medium with or without 2.5 mM STZ. Nearly all NSCs and differentiating cells express the IR (NSCs: approximately 96%; differentiating cells: approximately 98%; Figure 6B) as well as the GLUT3 (NSCs: approximately 91%; differentiating cells: approximately 95%; Figure 6D) without STZ treatment. However, STZ treatment reduced the number of NSCs in general as well as the percentage of IR-positive NSCs by 42.2% (*p* = 0.003; Figures 6A,B) and GLUT3-positive NSCs by 61.7% (*p* = 0.001; Figures 6C,D) compared to the control group. Different from the effect of STZ on NSCs, STZ did not affect the percentage of IR-positive differentiating cells (Figures 6A,B). But, treatment of differentiating cells with STZ resulted in a significantly decreased percentage of GLUT3-ir cells by 47.3% compared to controls (*p* = 0.015; Figures 6C,D).

## Discussion

STZ-treated adult NSCs produce fewer and smaller neurospheres as well as a reduced percentage of BrdU-positive cells. Although we could not exclude the possibility that the decreasing number and size of neurospheres mainly are the result of cell death, the decreased number of BrdU-positive cells indicate that at least the inhibition of cell proliferation is partly participating in the reduction of NSCs numbers. These findings are similar to a study previously reported by Qu et al. (2012), even if they used a much higher concentration of STZ (8 mM) than we did. As shown by Qu et al. (2012) STZ elicits a striking increase of cellular reactive oxygen species (ROS) in NSCs. Although proliferative NSCs maintain high endogenous ROS status and pharmacological or genetic manipulations that diminished cellular ROS levels interfered with normal NSCs function both in *in vitro* and *in vivo* studies (Le Belle et al., 2011), an excess of intracellular ROS induces cell death and inhibits NSCs proliferation (Limoli et al., 2006). Elevated intracellular ROS levels in NSCs caused by the treatment of NSCs with the glutathione synthetase inhibitor buthionine sulfoximine reduces the number of NSCs (Prozorovski et al., 2008).

Different from the results of this *in vitro* study, our previously published *in vivo* study (Sun, 2015; Sun et al., 2015) suggested that STZ significantly affects the survival of newborn cells, but not stem cell proliferation (Sun, 2015; Sun et al., 2015). The micro-environment in an animal’s brain is much more complex than in cell culture and this may be the reason for the discrepancy between *in vivo* and *in vitro* study results. *In vivo*, STZ may not only target newborn cells directly (as it certainly happens in cell culture) but also indirectly through acting on other types of cells. *In vivo*, the neurogenic niche, where NSCs give birth to new cells, contains various cell types such as astrocytes, microglial cells, various types of neurons as well as endothelial cells (as NSCs are localized in close proximity of blood vessels) and all these cells form a complex neurogenic micro-environment in the subgranular zone (Palmer et al., 2000). In consequence, the neurogenic niche plays an important role in the regulation of the survival and self-renewing capacity of stem cells (Kazanis et al., 2008) that depends on the change of vasculature (Palmer et al., 2000), growth and trophic factors (Anderson et al., 2002; Lee et al., 2002) and the support through glial cells (Morrens et al., 2012). A comparable micro-environment for proliferating NSCs is almost missing in a normal cell culture system, as in *in vitro* studies. Besides directly affecting NSCs STZ could also induce neuronal apoptosis (Unsal et al., 2016), astrogliosis and the activation of microglia (Chen et al., 2013). Therefore, NSCs in the brain could be regulated by several factors secreted by apoptotic neurons or activated microglia, such as high mobility group box 1 (HMGB1) and TNF-α, respectively (Kawabata et al., 2010; Shu et al., 2018). Inhibition of cells secreting proinflammatory cytokines (i.e., IL-1β, IL-6, TNF-α and IFN-γ) significantly inhibited neurogenesis in the subventricular zone (Shigemoto-Mogami et al., 2014). Furthermore, AN is shown to be remarkably influenced by several growth and trophic factors such as insulin/IGF (Grünblatt et al., 2007), NGF (Hellweg, 1994) as well as BDNF (Shonesy et al., 2012; Liu et al., 2014) which were suggested to be altered in the STZ icv rat model. We speculate that the discrepancy of results revealed by our recently published *in vivo* study and this cell culture study may be primarily due to the complex micro-environment of an AN niche missing under *in vitro* conditions. Furthermore, although STZ treatment could impair NSCs proliferation directly, the interactions between NSCs, mature neurons and glia cells may compensate this damage in the animal’s brain.

Another reason of this discrepancy may derive from different methods for analysis applied in these studies. In our *in vivo* study, we detected stem cell proliferation and the number of newborn cells of the neuronal lineage by immunostaining of the endogenous markers MCM2 and NeuroD, respectively. Newborn cells survived for almost 4 weeks were analyzed with the BrdU integration and detection method. We revealed that 1 month after STZ treatment stem cell proliferation is not affected, but that 3 months after STZ treatment the number of survived BrdU-ir cells was significantly decreased. In the *in vitro* study, however, we counted newly produced neurospheres and BrdU-positive cells after a various number of days of STZ incubation. These different methods applied and different time lines may also induce discrepancies between *in vivo* and *in vitro* studies.

Migration of NSCs is a prerequisite for the formation of the central nervous system (CNS) and also plays a pivotal role in AN in the CNS of mammals (Hatten, 1999). Molecular mechanisms involved in the migration of NSCs in the adult brain are still poorly understood. Neural progenitors transplanted into mouse brain migrate towards areas of brain damage resulting from stroke (Arvidsson et al., 2002) or glioblastoma (Glass et al., 2005). Such studies support the idea that tumor necrosis factor-α (TNF-α), interferon-γ (IFN-γ) and monocyte chemoattractant protein-1 (MCP-1; Belmadani et al., 2006) secreted by damaged brain areas regulate the migration of these differentiating NSCs towards sites of inflammation. Moreover, it has been shown that STZ icv treatment elevates the level of TNF-α in rat brain (Rai et al., 2014) and IFN-γ in peripheral blood lymphocytes (Pandey and Bani, 2010). Whether these elevated TNF-α and IFN-γ levels then impact the migration of differentiating newborn cells is still an open question. Our *in vitro*-study showed that STZ did not influence the migration distance and migration speed of newborn cells. However, in our study we measured the migration of all kinds of cells (e.g., immature neurons and astrocytes) and cannot provide information about the migration performance of a specific cell type.

Contrary to our *in vivo* results (Sun, 2015; Sun et al., 2015), STZ in cell culture seems to influence the differentiation fate of newborn cells. We used a 2 days differentiation paradigm (cells were kept for 2 days in differentiation medium), which is shown to mimic the initial stage of differentiation with differentiating cells already expressing Tuj1 and GFAP (Aranha et al., 2011). Qu et al. (2012) also found that STZ reduces neuronal differentiation of NSCs using three neuronal markers, Tuj-1, microtubule-associated protein 2 (MAP 2) and neurofilament 150 (NF 150; Qu et al., 2012). Our data show that after 2 days of differentiation 15% of cells were Tuj-1 positive, and this percentage is lower compared to the results of Dong (34%, incubation in differentiation medium for 3 weeks; Dong C. et al., 2012) and of Qu (20%, incubation in differentiation medium for 1 week; Qu et al., 2012). However, besides different incubation times in differentiation medium applied in these studies, they used rats of different ages (postnatal day 0 or embryonic day 17 rats) for the isolation of NSCs. Because we studied AN, we selected young adult rats for this research. Our study showed that the percentage of GFAP-ir cells (20%) did not change in consequence of STZ treatment. Like with the proliferation of NSCs (we already discussed above) ROS may also impact the differentiation of newly produced cells. As high ROS levels seem to reduce new neuron generation, e.g., with differentiation of NSCs for 7 days in the presence of pro-oxidative bothionine sulfoximine or diethyldithiocarbamate, ROS seem to contribute to neural-fate decision (Prozorovski et al., 2008).

Under normal conditions, nearly all NSCs express IR and GLUT3 proteins. Although STZ treatment decreased number of NSCs (Figure 2F), remaining cells exhibit even a lower percentage of cells expressing IR and GLUT3 protein. In contrast to the staining results with antibodies detecting IR protein, mRNA expression levels of this receptor were not found to be influenced by STZ. Therefore, STZ may influence the process of IR protein translation, post-translational modification and/or subcellular distribution. As many studies have shown, Insulin/PI3 kinase signaling is necessary to maintain NSCs survival and self-renewal in the adult brain (Groszer et al., 2006; Siegrist et al., 2010).

Glucose transport into adult NSCs mainly relies on GLUT1 and GLUT3 as they do not express GLUT2 and GLUT4 (Maurer et al., 2006). The expression of GLUT1 is relatively stable. However, GLUT3 in NSCs seems to be sensitive to environmental changes. Stress induced by hypoxia and/or hyperglycemia dramatically increases GLUT3 expression at both, protein and mRNA levels, but only slightly up-regulates GLUT1 protein levels in NSCs (Maurer et al., 2006). There exist only few indications in the literature for a relationship between NSCs’ proliferation and GLUT3 expression. However, GLUT3 was found to promote tumor cell proliferation in non-small cell lung cancer (Masin et al., 2014). GLUT3 may also increase NSCs’ proliferation through the transport of more glucose into NSCs enhancing energy supply.

Differentiation fate of newborn cells may also be affected by the expression of GLUT3. At the stage of NSCs both transporters, GLUT1 and GLUT3, are expressed (Maurer et al., 2006), however, in astrocytes only GLUT1 can be detected, whereas newborn neurons mainly depend on GLUT3 for glucose transport (McCall et al., 1996; Dienel, 2012). The level of GLUT3 and GLUT1 expression may influence the potential of NSCs to differentiate. Decreased GLUT3 expression caused by STZ treatment may thus reduce the potential of NSCs toward neuronal-oriented differentiation without affecting the differentiation to astrocytes.

In summary, our *in vitro* study showed that STZ influences AN at different stages, during NSCs’ proliferation as well as during differentiation of their progeny. More detailed, 2.5 mM STZ inhibits the proliferation of NSCs in a dose/time-dependent manner and impairs new neuron generation but not the production of new astrocytes. Furthermore, STZ remarkably affects the expression of metabolism-related genes/proteins such as GLUT3. All these attempts will help to further highlight the role of AN in the etiopathogenesis of dementia and especially of sAD with the overall aim to unravel factors and mechanisms for the treatment of sAD. Beyond that, we hope that cultured NSCs analyzed in this study could be used as a cell model to screen new compounds for the treatment of AD and other aging-related diseases. Establishing a co-culture system would help to improve the study of STZ effects on NSCs as co-culturing of NSCs with multiple other cell types would help to overcome the missing micro-environment influencing STZ treatment. Moreover, rats of 2 months of age used for this *in vitro* study are young adult animals and certainly do not have the best age to study neurobiological mechanisms underlying a neurodegenerative disorder such as sAD. Therefore, using neurospheres derived from 2 months old animals are a limitation of this study and we will use NSCs derived from older animals in the future.

## Author Contributions

PS and AS-B designed the experiments. PS and GO performed the experiments and analyzed data. PS, YT, QH, PR, JD and AS-B discussed and interpreted the results. PS and AS-B wrote the article. All authors have approved the final version of the manuscript.

## Conflict of Interest Statement

The authors declare that the research was conducted in the absence of any commercial or financial relationships that could be construed as a potential conflict of interest.
